# Inflammatory hypothesis of atherogenesis: Will colchicine be added to the armamentarium in the prevention of coronary artery disease?

**DOI:** 10.1016/j.ahjo.2021.100057

**Published:** 2021-10-07

**Authors:** Bahadir Simsek, Bugra Han Egeli, Atakan Selte, Bibin Varghese, Roger S. Blumenthal, Erin Chew

**Affiliations:** a Minneapolis Heart Institute Foundation, Minneapolis, MN, USA; b Postgraduate Medical Education, Harvard Medical School, Boston, MA, USA; c Department of Pediatrics, Children's Hospital of Los Angeles, University of Southern California, CA, USA; d University of Wisconsin-Madison, Department of Neurology, Madison, WI, USA; e Ciccarone Center for the Prevention of Cardiovascular Disease, Johns Hopkins University, Baltimore, MD, USA; f Division of Cardiovascular Medicine, Division of Medicine, Vanderbilt University, Nashville, TN, USA; g Division of Rheumatology, Department of Medicine, Vanderbilt University, Nashville, TN, USA

**Keywords:** Atherosclerosis, Cardiovascular diseases, Colchicine, Coronary artery disease, Inflammation

## Abstract

Chronic inflammation promotes the development and progression of atherosclerosis. Despite aggressive risk reduction, patients with coronary artery disease have a significant residual risk of myocardial infarction and cardiovascular death related in part to ongoing inflammation within coronary vasculature. In this review, we summarize the clinical trials that provide evidence for the inflammatory hypothesis of atherogenesis. Additionally, we describe studies suggesting colchicine may be able to reduce residual inflammatory risk via the NLRP3 pathway. Given its tolerable side effect profile, safety, and low cost, colchicine holds promise as an anti-inflammatory agent in primary and secondary prevention of coronary disease.

## 1 Introduction

Atherosclerotic cardiovascular disease (ASCVD) remains the leading cause of morbidity and mortality worldwide [1]. Despite aggressive risk reduction using lipid-lowering and antihypertensive therapy, patients with coronary artery disease (CAD) have a significant residual risk of myocardial infarction (MI) and cardiovascular death. Although abnormal cholesterol deposition within the vascular endothelium initiates the process of plaque formation, ongoing low-grade inflammation within the vascular endothelium may lead to plaque progression and destabilization and partially explain the phenomena of residual risk [2].

The Canakinumab Anti-Inflammatory Thrombosis Outcome Study (CANTOS) was the first landmark trial that validated the inflammatory hypothesis in atherosclerosis, where interleukin-1β (IL-1β) antagonism was shown to decrease major adverse cardiovascular events (MACE) independent of lipid-lowering [3]. Thereafter, the Colchicine Cardiovascular Outcomes Trial (COLCOT) and Low-Dose Colchicine 2 (LoDoCo2) trials highlighted the potential therapeutic role of colchicine in secondary prevention [4], [5]. These trials highlight the fact that inflammatory pathways may serve as potential therapeutic targets in reducing residual risk.

This review summarizes the inflammatory hypothesis of atherosclerosis, landmark trials that confirmed the role of inflammation in ASCVD, and describes ongoing studies for the prevention and treatment of atherosclerosis (Table 1).

**Table 1:** Major trials testing the inflammatory hypothesis of atherogenesis, and the efficacy of colchicine in the secondary cardiovascular disease prevention.

First author, trial name, year, n	Participants and age	Intervention	Clinical outcomes	Results
Ridker PM, CANTOS, 2017 n = 10,061	Patients with previous MI and a hs-CRP level of 2 mg or more per liter Mean Age: 61	Canakinumab (50 mg, 150 mg, and 300 mg), administered subcutaneously every 3 months **Follow up:** 3.7 years	**Primary outcome:** the first occurrence of nonfatal MI, any nonfatal stroke, or CV death. **Secondary endpoint:** components of the primary end-point, hospitalization for unstable angina that led to urgent revascularization.	Significant reduction in the primary endpoint (p = 0.02), key secondary CV endpoint (p = 0.003). MI, stroke or death from any cause (p = 0.02), hospitalization for unstable angina that led to urgent revascularization (0.005). No significant difference in all-cause mortality (p = 0.31).
Ridker PM, CIRT, 2019 n = 4786	Patients with previous MI or multivessel coronary disease who additionally had either type 2 diabetes or the metabolic syndrome Mean Age, **Intervention:** 65.6 (59.7–71.8) **Placebo:** 66.0 (59.8–71.7)	Low-dose methotrexate 15–20 mg per week **Follow up:** 2.3 years	**Primary endpoint:** a composite of nonfatal MI, nonfatal stroke, or CV death, hospitalization for unstable angina that led to urgent revascularization **Secondary endpoints:** death from any cause, MACE, hospitalization for congestive HF	No significant reduction in the primary endpoint (HR = 0.96, 95% CI = 0.79–1.16, p = 0.67). No significant reduction in the secondary endpoints. No significant reduction in the tertiary endpoints. No detection of lower interleukin-1β, interleukin-6, or CRP levels than placebo.
Deftereos S, Colchicine Treatment for the Prevention of Bare-Metal Stent Restenosis in Diabetic Patients, 2013, n = 196	Patients with diabetes with contraindication to DES undergoing PCI with BMS Mean Age, Intervention: 63.7 ± 6.9 Placebo: 63.5 ± 7.2	Colchicine 0.5 mg twice daily Follow up: 6 months	**Primary outcome:** Angiographic and IVUS-defined ISR. **Secondary outcome:** Angiographic and IVUS-defined lumen loss and in-stent neointimal hyperplasia	Significantly lower angiographic ISR (OR = 0.38, 95% CI = 0.18–0.79, p = 0.007). Significantly lower IVUS-defined ISR (OR = 0.42, 95% CI = 0.22–0.81). Significantly lower lumen area loss 1.6 mm^2^ vs 2.9 mm^2^ (p = 0.002).
Nidorf SM, LoDoCo, 2013 n = 532	35–85 year old patients with angiographically proven coronary disease Mean age, **Intervention:** 66 ± 9.6 **Placebo:** 67 ± 9.2	Colchicine 0.5 mg/day Follow up: 3 years	Composite incidence of acute coronary syndrome, out-of-hospital cardiac arrest, or non-cardioembolic ischemic stroke	Significant decrease in the occurrence of primary outcome in the colchicine group (HR = 0.33, 95% CI = 0.18–0.59, p < 0.001).
Tardif JC, COLCOT, 2019 n = 4745	Adult patients that had a MI within 30 days before enrollment, completed any planned percutaneous revascularization procedures, and had been treated according to national guidelines Mean age, **Intervention:** 60.6 ± 10.7 **Placebo:** 60.5 ± 10.6	Colchicine 0.5 mg/day **Follow up:** 22.6 months	**Primary end point:** a composite of death from CV causes, resuscitated cardiac arrest, MI, stroke, or urgent hospitalization for angina leading to coronary revascularization **Secondary end points:** the components of the primary efficacy end point	Significant decrease in the occurrence of primary outcome in the colchicine group (HR = 0.77, 95% CI = 0.61–0.96, p = 0.02). No significant difference in death from CV causes, resuscitated cardiac arrest, MI, stroke rates and urgent hospitalization for angina leading to coronary revascularization in the colchicine group. Significantly higher pneumonia rates in the colchicine group (p = 0.03).
Shah B, COLCHICINE-PCI, 2020 n = 400	Patients aged ≥18 years with suspected ischemic heart disease or acute coronary syndromes referred for clinically indicated coronary angiography with possible PCI Mean age, **Intervention:** 65.9 ± 9.9 **Placebo:** 66.6 ± 10.2	Colchicine 1.8 mg, administered 1–2 h before the procedure Follow up: 30 days	**Primary outcome:** PCI related myocardial injury **Secondary outcome:** occurrence of 30-day MACE, a composite of the earliest occurrence of death from any cause, nonfatal MI, or target vessel revascularization	No significant difference in the percutaneous coronary intervention-related myocardial injury (p = 0.19) or 30-day MACE (p = 0.82), PCI-related MI (p = 0.49), type 1 MI (p = 0.49), type 4a MI (p = 0.89), all-cause mortality (p = 0.99) and target vessel revascularization at 30 days.
Nidorf SM, LoDoCo2, 2020 n = 5522	Patients 35 to 82 years of age that had any evidence of coronary disease on invasive coronary angiography or computed tomography angiography Mean age, **Intervention:** 65.8 ± 8.4 **Placebo:** 65.9 ± 8.7	Colchicine 0.5 mg/daily **Follow up:** 28.6 months	**Primary composite outcome:** CV death, spontaneous (non-procedural) MI, ischemic stroke, or ischemia-driven revascularization **Secondary outcome:** components of the primary endpoint, death from any cause	Significantly primary end-point events in the colchicine group (HR = 0.69, 95% CI = 0.57–0.83, p < 0.001). Significantly reduced secondary end-point events in the colchicine group; including CV death, spontaneous MI, and ischemic stroke (p = 0.007). No significant difference in death from any cause. Higher incidence of death from non-CV causes in the colchicine group.
Tong DC, COPS, 2020 n = 795	Patients (18–85 years) who presented with ACS and had evidence of coronary artery disease on coronary angiography **Mean age:** 59.8 ± 10.3	Colchicine 0.5 mg twice daily for the first month, followed by 0.5 mg daily for another 11 months **Follow up:** 12 months	**Primary outcome:** the composite of all-cause mortality, ACS, ischemia leading to urgent coronary revascularization, and non-cardioembolic ischemic stroke	No significant difference in the primary outcome (HR = 0.65, 95% CI = 0.38–1.09, p = 0.09). Higher rate of death in the colchicine group (p = 0.018). Higher rate of non-CV death in the colchicine group (p = 0.024).
PI: Sanjit S Jolly, MD CLEAR SYNERGY (OASIS-9), Estimated n = 7000	Patients 18 years or older, with STEMI who have undergone PCI	Colchicine 0.5 mg twice daily Spironolactone 25 mg daily	**Primary endpoint:** MACE, composite of CV death, recurrent MI, or stroke, composite of CV death or new or worsening HF	(ongoing)
PI: Prof. Peter Kelly, CONVINCE, Estimated n = 2623	Patients over 40 years of age who have suffered an ischaemic stroke or transient ischaemic attack not caused by cardiac embolism or other defined causes	Colchicine 0.5 mg daily	Recurrence of non-fatal ischemic stroke, non-fatal MACE, non-fatal hospitalization for unstable angina, MI, cardiac arrest, vascular death	(ongoing)

ACS: Acuter coronary syndrome, BMS: Bare metal stent, CV: Cardiovascular, DES: Drug eluting stent, HF: Heart failure, IVUS: Intravascular ultrasound, hs-CRP: high-sensitivity C-reactive protein, MACE: Major adverse cardiac events, MI: Myocardial infarction, PCI: Percutaneous coronary intervention, STEMI: ST-elevation myocardial infarction.

## 2 Role of inflammation in development of atherosclerosis

Plaque formation begins with endothelial injury and subsequent deposition of LDL-cholesterol (LDL-C) within the subintimal space. Oxidized LDL stimulates the innate and adaptive immune system inducing the expression of adhesion molecules, chemoattractants, and growth factors. A variety of immune cells, mainly monocytes and macrophages, but also CD4^+^ T cells are recruited to early atherosclerotic plaque sites. Monocytes turn into macrophages, phagocytose the lipid particles, and form foam cells, which further set off sequelae of the inflammatory cascade. Subsequent smooth muscle cell proliferation and the formation of extracellular matrix entraps plasma lipids giving rise to a lipid-rich necrotic core of plaques [6].

**Fig. 1:**
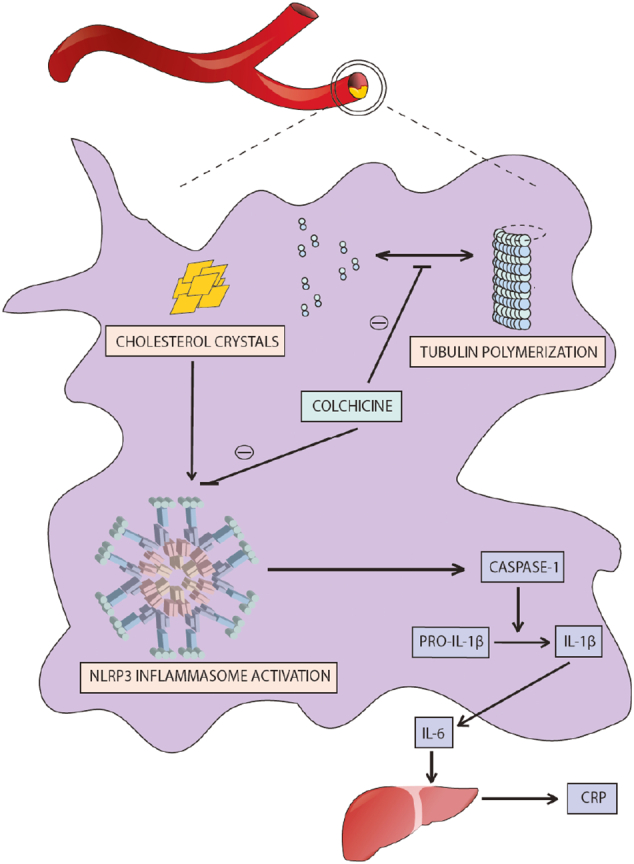
Mechanisms of action of colchicine in white blood cells.

Saturated lipid cores lead to cholesterol crystallization, which further propagates the inflammatory response via inflammasomes, complex cytosolic supramolecules critical to host immune defense. Cholesterol crystals stimulate proinflammatory macrophages to assemble the Nod-like receptor protein (NLRP3) inflammasome within the plaque. Activated NLRP3 recruits caspase-1, which cleaves pro-interleukin-1β into its mature form, IL-1β. Once activated, IL-1β stimulates a strong inflammatory response (including the production of interleukin-6 (IL-6) and C-reactive protein (CRP) within the endothelium, ultimately leading to cell death and plaque destabilization) (Fig. 1) [6].

## 3 Anti-inflammatory therapies

### 3.1 Statins

Statins inhibit HMG-CoA reductase, the rate-limiting enzyme in cholesterol synthesis, and this leads to upregulation of LDL receptors on the hepatocytes. Substantial evidence has confirmed the causal link between the lipid-lowering effects of statin therapy to the reduction of MACE. However, statins also have pleiotropic anti-inflammatory effects independent of lipid-lowering [7]. The anti-inflammatory and antioxidative effects of statins are proposed to work through the suppression of oxidation pathways such as the inhibition of NADPH oxidase, myeloperoxidase, and the prevention of nitric oxide-derived oxidants [8]. The magnitude of the benefit associated with statin therapy is thought to correlate with reductions in inflammatory markers.

In JUPITER, a randomized placebo-controlled trial including patients with normal LDL-C (130 mg/dL) and elevated high-sensitivity CRP (hsCRP) (>2 mg/L), 20 mg of rosuvastatin reduced hsCRP levels by 37%, and significantly reduced incidence of MACE and cardiovascular death by about 45%, (p < 0.00001) [7], which is more than that can be accounted for by the lipid lowering effects of statins [7]. Based on these results, elevated hsCRP is listed as a risk-enhancing factor in estimating ASCVD risk per the 2019 ACC/AHA Guideline on the Primary Prevention of Cardiovascular Disease [9].

### 3.2 Canakinumab

Interleukin-1β is a proinflammatory cytokine and strong inducer of innate immunity. Activated NLRP3 results in the production of IL-1β, thereby amplifying the inflammatory cascade that promotes atherosclerosis progression and plaque destabilization [10]. Canakinumab is an anti-IL-1β monoclonal antibody currently approved for several rare rheumatological conditions (including cryopyrin-associated periodic syndromes, refractory gout, and Muckle–Wellis syndrome), where increased inflammasome activity and production of IL-1β are thought to drive disease activity [11].

The Canakinumab Anti-inflammatory Thrombosis Outcome Study (CANTOS) trial showed that Canakinumab significantly reduces inflammatory markers without major effects on LDL-C or HDL—C. In a phase IIb study, 556 men and women with high cardiovascular disease risk and well-controlled diabetes mellitus were randomized to canakinumab or placebo. At the end of the 4-month follow-up, canakinumab significantly reduced hsCRP and IL-6 levels in a dose response relationship, without affecting hemoglobin A1c, LDL-C, HDL—C, or non–HDL-C levels [12].

In the phase III portion of the CANTOS trial, 10,061 patients with a history of an MI and a median hsCRP level of 2 mg/L or more on optimal cardiovascular medical treatment were enrolled. The participants randomized to 50, 150, and 300 mg of subcutaneous canakinumab every 3 months had a reduction in hsCRP levels by 26%, 37%, 41%, respectively. The group receiving 150 mg of canakinumab had a significant 17% risk reduction in nonfatal MI, nonfatal stroke and cardiovascular death.

Notably in a subgroup analysis, those with levels of hsCRP < 2 mg/L after 3 months of therapy had a 31% reduction in cardiovascular mortality (p = 0.0004), and all-cause mortality (p < 0.0001). Those who reached lower IL-6 levels after canakinumab treatment had a 32% reduction in MACE. However, canakinumab was associated with more fatal infections or cases of sepsis compared to placebo [3]. The CANTOS trial was the first study to demonstrate that lowering inflammation can reduce MACE independent of lipid levels.

### 3.3 Methotrexate

Methotrexate is a folic acid antagonist used for the treatment of a variety of inflammatory rheumatologic conditions. Given prior evidence of cardiovascular benefit of methotrexate in patients with rheumatoid arthritis [13], [14], the Cardiovascular Inflammation Reduction Trial (CIRT) hypothesized that the use of low-dose methotrexate might result in lower cardiovascular event rates in those with established CAD. Patients over the age of 18 years with MI or multivessel coronary disease in addition to type 2 diabetes mellitus or metabolic syndrome were randomized to either low-dose methotrexate or placebo [15].

Methotrexate did not significantly reduce rates of nonfatal MI, nonfatal stroke, or cardiovascular death when compared to placebo (HR, 0.96; 95% confidence interval, 0.79–1.06). Of note, low-dose methotrexate did not lead to lower IL-1β, IL-6, or CRP compared to placebo. The results of the CIRT suggested that inhibition of IL-1β, IL-6, and CRP levels likely is necessary for atherosclerotic protection and reduction in cardiovascular events.

### 3.4 Colchicine

Colchicine has been routinely used in the treatment of several rheumatological conditions such as gout and familial Mediterranean fever (FMF) [16]. Colchicine irreversibly binds alpha and beta-tubulin subunits, thereby blocking microtubule polymerization, which is required for intracellular signaling, mitotic spindle formation, mitosis and cell migration [17], [18]. Colchicine thereby affects any cellular process that requires cytoskeletal rearrangement, including neutrophil motility, platelet activation and aggregation, and proliferation of smooth muscle cells [16], [19].

Additionally, by inhibiting intracellular signaling, colchicine is known to prevent NLRP3 inflammasome assembly, thereby preventing the release of IL-1β and other proinflammatory cytokines [20], [21]. Independent of tubulin binding, colchicine further impairs platelet-leukocyte interaction, decreases activation and adhesion of T lymphocytes, and reduces selectin expression on inflamed vascular endothelium [22].

## 4 Colchicine in the secondary prevention of coronary artery disease

Three RCTs suggest that the anti-inflammatory properties of colchicine can be used as a secondary prevention strategy in patients with CAD. In the Low Dose Colchicine for Secondary Prevention of Cardiovascular Disease (LoDoCo) RCT, investigators enrolled 532 patients between the ages of 35–85 with stable CAD treated with aspirin and statin therapy and randomized them to either receive colchicine 0.5 mg/day or placebo. After a median follow-up period of 3 years, colchicine reduced the primary outcome of acute coronary syndrome (ACS), cardiac arrest, and stroke by 67%. Therefore, in this trial, colchicine was efficacious for the secondary prevention of cardiovascular events in patients with stable CAD [23].

The Colchicine Cardiovascular Outcomes Trial (COLCOT) enrolled 4745 patients within 30 days after a MI, and randomized them to receive colchicine (0.5 mg/day) or placebo. In this double-blind RCT, after a median follow-up of 22.6 months, colchicine therapy reduced the rate of the primary outcome (composite death from cardiovascular causes, resuscitated cardiac arrest, MI, stroke, or urgent hospitalization for angina leading to coronary revascularization) by 23% when compared to placebo. While no significant difference was detected in death from cardiovascular causes, resuscitated cardiac arrest, or MI; stroke rates and urgent hospitalization for angina leading to coronary revascularization were significantly lower in the colchicine group. Overall, there was no significant difference in serious adverse events between the groups. However, pneumonia rates were significantly higher in the colchicine group (21/2330) compared to placebo (9/2346), (0.9% vs 0.4%, p = 0.03).

The authors concluded that colchicine significantly lowered the cardiovascular event rates compared to placebo among patients with recent MI [5]. In a follow-up study, investigators aimed to determine whether time-to-treatment initiation (≤3, 4–7, ≥8 days) influenced the beneficial effects rendered by colchicine. After a median follow-up of 23 months, patients in whom colchicine was initiated ≤3 days after MI had a significant reduction in the primary end-point events compared to placebo (HR = 0.52; 95% CI 0.32–0.84) as well as significant improvements in urgent hospitalization for angina requiring revascularization (HR = 0.35), all-coronary revascularization (HR = 0.63), and the composite of MI, stroke, resuscitated cardiac arrest, or cardiovascular death (HR = 0.55, p < 0.05). The clinical benefits were not seen in patients who were started on colchicine 4 or more days after MI [24].

These results are consistent with a pilot RCT of 150 people who were enrolled within 12 h of ST-elevation myocardial infarction (STEMI) and randomized to colchicine or placebo [25]. Those who were given colchicine for 5 days immediately after STEMI had significantly lower levels of CK-MB (p < 0.001), as well as significantly smaller infarct sizes on gadolinium-enhanced MRI (p = 0.019) [25]. Therefore, early initiation of colchicine after a MACE may be important for maximizing its protective effects.

Based on the results of the LoDoCo trial and COLCOT trial, investigators performed another RCT Low-Dose Colchicine-2 (LoDoCo2) to evaluate the cardiovascular risk reduction effects of colchicine in patients with chronic coronary disease. In this double-blind RCT of 5522 patients, colchicine therapy reduced the incidence of the primary composite outcome (cardiovascular death, spontaneous (non-procedural) MI, ischemic stroke, or ischemia-driven revascularization) by 31% when compared to placebo. Colchicine also significantly reduced the secondary end-point events, which included cardiovascular death, spontaneous MI, and ischemic stroke. However, there was a non-significant increase in the incidence of non-cardiovascular deaths (HR = 1.51, 95% CI 0.99–2.31). The authors concluded that in patients with chronic coronary disease, 0.5 mg daily colchicine significantly reduced the risk of nonfatal cardiovascular events compared to placebo [4].

Despite these studies demonstrating significant benefits for the use of colchicine in the prevention of MACE, several studies suggested no benefit or potential harm. In COLCHICINE-PCI, investigators enrolled 400 participants referred for PCI. In this trial, 1–2 h preprocedural administration of 1.8 mg colchicine did not reduce the percutaneous coronary intervention (PCI)-related myocardial injury or 30-day major cardiovascular composite events of death, nonfatal MI, and target vessel revascularization. While colchicine did not result in a significant change in IL-6 concentrations at 1-h post-PCI, the increase in IL-6 and hsCRP were significantly lower at 24-h post-PCI compared to placebo. This study has limitations such as the acute nature of the procedure allowing only 1–2 h of pre-procedural colchicine, and the short-term timepoints, which could have led to the non-significant difference [26].

In the Colchicine in Patients with Acute Coronary Syndrome (COPS) multicenter double-blind, placebo-controlled RCT, 17 hospitals across Australia enrolled 795 patients between the ages of 18–85 who presented to the hospital with ACS and had evidence of CAD on coronary artery angiography treated either by percutaneous coronary intervention or medical therapy alone. Patients were randomized to colchicine (0.5 mg twice daily for the first month, followed by 0.5 mg daily for another 11 months) or placebo. After 12-months of follow-up, colchicine therapy led to a non-significant reduction in the incidence of the primary outcome (all-cause mortality, ACS, ischemia leading to urgent coronary revascularization, and non-cardioembolic ischemic stroke) when compared to placebo (24 vs 48 events, p = 0.09, log-rank).

While there were 8 deaths in the colchicine group, there was only a single death in the placebo group. Reported adverse effects were similar between the groups with 23% in the colchicine group, and 24.3% in the placebo group. As a result, in this select group of patients the addition of colchicine to standard therapy did not improve cardiovascular outcomes, and led to a higher incidence of total death [27].

The accompanying editorial highlighted that the primary composite outcome was in favor of colchicine (p = 0.65; 95% CI 0.38–1.09) but did not reach statistical significance, possibly because there were only 62 primary endpoint events in this small trial. Reported deaths in this trial (8 in colchicine vs 1 in placebo) must be interpreted cautiously considering that loss to follow-up in this study was twice the reported deaths. In addition, this editorial highlighted that the increased non-cardiovascular death trend observed in the COPS trial is inconsistent with the previous COLCOT and LoDoCo2 trials as well as safety data from previous trials of colchicine in patients with gout, FMF, and pericarditis [28].

Preliminary evidence suggests that colchicine may also have cardiovascular benefits when used as primary prevention. A recent study of 722 patients without known CAD and gout demonstrated that colchicine administration was associated with 63% lower rates of incident MI, evidence of ischemia, or obstructive CAD on stress test or angiography [29], [30]. Although this was a non-randomized study with a small sample size, this study could pave the way for new studies investigating whether colchicine can lower CVD events when used in primary prevention.

## 5 Conclusion

Inflammation plays a pivotal role in the pathogenesis of ASCVD. Targeting inflammatory pathways have significant potential to intensify prevention strategies, especially in high risk patients. CANTOS and several colchicine studies (COLCOT, LoDoCo, LoDoCo2) have confirmed the inflammatory hypothesis of coronary disease and demonstrated that targeting residual inflammatory risk and possibly the NLRP3 pathway may serve as an important strategy in reducing major adverse cardiovascular events. It will be necessary to get FDA approval or the inclusion of the therapy into AHA/ACC Clinical Practice Guidelines, however, before this can be recommended.

Given its tolerable side effect profile, safety, and low cost, colchicine holds promise as an anti-inflammatory agent in reducing cardiovascular events. However, the unexpected increase in non-cardiovascular death in the COPS trial is a concern and will need further study. The ongoing RCTs such as Colchicine and Spironolactone in Patients with MI/SYNERGY Stent Registry (CLEAR SYNERGY) and Colchicine for Prevention of Vascular Inflammation in Non-cardio Embolic Stroke (CONVINCE) trials will add to the current knowledge by testing colchicine in patients with MI undergoing PCI, and in patients who had previous ischemic stroke or transient ischemic attack attributed to atherosclerosis respectively.

## Funding

None.

## CRediT authorship contribution statement

Bahadir Simsek: Conceptualization, manuscript drafting and critical revision, visualization. Bugra Han Egeli: Conceptualization, manuscript drafting and critical revision, visualization. Atakan Selte: Conceptualization, manuscript drafting and critical revision, visualization. Bibin Varghese: Conceptualization, manuscript drafting and critical revision. Roger S. Blumenthal: Conceptualization, manuscript drafting and critical revision, supervision. Erin Chew: Conceptualization, manuscript drafting and critical revision.

## Declaration of competing interest

The authors declare that they have no known competing financial interests or personal relationships that could have appeared to influence the work reported in this paper.
